# Valedictory Address for the Graduating Class

**Published:** 1878-05

**Authors:** George W. Blanton

**Affiliations:** Dalton, Ga.


### VALEDICTORY ADDRESS FOR THE GRADUATING CLASS IN ATLANTA MEDICAL COLLEGE, MARCH 6, 1878.

By GEORGE W. BLANTON, M.D., Dalton, Ga.

*Honored Professors, Fellow Students, Ladies and Gentleman:*

By the kind partiality of my comrades, I have been honored with the duty of speaking a farewell word on this occasion.

You, my fellow-students, are aware of the difficulties which we have so far encountered. There was a time in my own experience, not long after beginning the study of medicine, when my untutored faculties were contendiug with the intricacies of anatomy, that I thought the obstacles to this occasion insuperable. But these initiatory difficulties, while only a faint indication of what is necessary for ultimate success, are at least in the past; and since they have been met and overcome, they are an omen of success in the future, if that future is marked by the same faithful effort that has characterized the past. As we stand to-night, on the threshold of practical life—more practical to the physician than others—it is but natural that bright anticipations of sickness—other people's sickness; of fees—paid by other people; of physic—swallowed by other people; and of—of *wives*—other people's daughters—should fill our minds.

To you, our honored educators, these difficulties of which we have been speaking appear as the toys possessed by the old man in childhood, and these bright anticipations as practical reminiscences of the past, or as stern realities of the present. We are indebted to you for your assistance in reference to the former, and beg you to lend us a helping hand in the latter.

The large audience with which Atlanta has honored this occasion, the liberal hospitality with which our stay here has been crowned, the many privileges she has extended to us, make us know that she has felt the deepest interest in our success and the permanent prosperity of our Alma Mater. We are proud to feel confident that her people would have sympathized with us in our day of probation to which we have alluded, and that she is thoroughly capable of appreciating the radiant hopes we cherish for the future. For one, I am proud of Atlanta, and I am sure that in so speaking I express the sentiments of every student of the Atlanta Medical College. We are proud that our Alma Mater is in Atlanta, with her energetic, intelligent people, her commerce and manufactories, her schools and her churches; that Atlanta is in Georgia, and that Atlanta is the capital of the State of Georgia, with her honest government, good laws, splendid climate, and productive lands, tilled by a thrifty, intelligent people; that Georgia is in the South, with her mineral, agricultural, social and religious advantages, such as are not enjoyed by any other people under the sun. I believe in that kind of patriotism which builds up home institutions, and I believe such a patriotism is the wisest of policies. As a commercial people, we can never attain the success we desire until home merchants and manufacturers are patronized. As an agricultural people, we shall never be properly enriched until our implements are manufactured at home, our products are sold here, worked into fabrics here, purchased here, and worn here. As a manufacturing people, we can never know our proper prosperity until our power is utilized, until many more of our enterprises are pushed by steam, and until we look no longer to the North for our implements. When Southern mines are worked by Southern capitalists; Southern fields are tilled with implements of Southern manufacture; Southern products are manufactured and worn by Southern people; and Southern institutions of learning are patronized by the brave young men and fair daughters of the South—then, AND NOT TILL THEN, will we ever reach the measure of prosperity which is ours by right, and assume that position in the government of national affairs, for the want of which we have suffered so much during the last decade.

Why should not such a thing be? I am sure it will be granted that our agricultural, manufacturing, mineral, social and religious advantages are as good as those of any other section of this continent; and if any doubt that the advantages of our literary, scientific, theological and medical institutions are not so good for fitting young men for the highest measure of usefulness and success, they have not taken the pains to investigate the subject, and are ignorant of the greatest blessing God has given us. I go a step farther, and maintain that for all that constitutes real success, if a young man is to live in the South, a Southern education better prepares him than one he could gain anywhere else, on this or any other continent. Any one who has observed much of the "eternal fitness of things," or the law of "cause and effect," and will give this proposition careful investigation, I am sure will agree with me. Taking it for granted, then, for the sake of argument, I ask, why is it that our institutions of learning do not have that patronage they deserve? Every thoughtful man must see the cause in those delusive dreams of some superior good which exists far from our homes. Distance lends enchantment to the swelling advertisements and fairy-like tales of the splendid advantages of institutions of other sections.

Lest some should mistake an indirect apology for an institution which can and does stand upon its own unrivalled excellence, for the point I am trying to make, I push it no further. Let none imagine that I am the apologist or eulogist of the Atlanta Medical College, for I have not been appointed to the task, and she needs not such an effort, even from the most gifted, since her equipments, her corps of professors and her previous history are her highest eulogies.

In this parting address, I do not feel that I would fully represent the sentiments of my comrades were I to fail to express to our beloved professors our warmest thanks for the efficient and untiring manner in which they have instructed us. We respect you for your ability, we honor you for your faithfulness, and we love you for your interest in us and your efforts in our behalf. It is our wish that your halls may be filled with students, your larders supplied with the fat of the land, your purses "burst out with new money," that you may never grow less in body or soul, "that you may live forever, and that we may never die." We shall go hence feeling that it was a privilege to be under your instruction, to feel the influence of the spirit that animates you, and we would be insensible to the powerful influence of example were we to leave these scenes, content with small measure of success. As we gaze far up the inclined plane that separates us, we are inspired with the motto, "Onward and Upward," for—

> "Lives of great men all remind us,
> We can make our lives sublime;
> And, departing, leave behind us,
> Footprints on the sands of time."

You and I, my fellow students, are to leave here to begin life in earnest; some of you may return, but some of us can say with the school girl, and with her meaning, "our school days are sadly o'er." As we enter the practice of our profession, we should be impressed with its importance. Every reason for virtue and intelligence that can apply to any calling, applies to ours. The nature of our work will invest us with extraordinary influence, and he who exercises great influence carries fearful responsibility. No man who refuses to see and feel this is fit for such a noble work. To a certain degree, the bodies of immortals are in the physician's keeping. In him is reposed the most sacred confidence of vital importance; to him are opened all the divinely appointed and most sacred secresy of family life. His presence is eagerly sought in scenes of sickness, suffering and death. His mission is to ameliorate the ills of life, and in his calling a well stored mind, an unblemished character, a strong will, an affable disposition, a kind and sympathizing heart, are among the many indispensables. The wonderful accumulations of knowledge in the science of medicine, the multiplied and multiplying facilities in its practice, demand ceaseless application to the study of the theory and practice to which our lives have been consecrated. We carry a part of the reputation of this institution upon our shoulders. Where we live she will be judged by our success or failure. Then let us be *worthy* of her. We will come in contact with the prejudice of many who practice a combination of ignorance and physic. Here we will need the wisdom of a serpent to protect the profession, and yet the harmlessness of a dove not to injure ourselves. I recently heard of a young *doctor* who had never attended a course of lectures, but was doing such practice as he could get. Being called into consultation with two physicians, he waited until they pronounced the patient "convalescing," whereupon he said, "Pshaw, *convalescence* is nothing; I've cured that many a time." His wisdom is only equaled by that of the school boy who wrote a composition on "pins," and said, among other wonderful things, that "pins had saved the lives of thousands of people." His teacher asked how, and the sage reply was: "Why, by not swallowing them." In the same manner I hope the medicine of the uninformed "quack" may save the lives of thousands.

The demands of social life, the capabilities of our mental faculties, the proper appreciation of the science of medicine, all call for general culture in the physician. He who boasts that he knows only medicine, does not know that. Away with such notions! Let the custodian of the lives of immortals utilize every source of information. Should he fail of his duty in this regard, how monstrous the crime!

> "Methought the billows spake and told me of it;
> The winds did sing it to me, and the thunder,
> That deep and dreadful organ-pipe, pronounced
> The name, 'Miscreant!'"

Were there a necessity I would insist that in our life-work, we should be inspired with a noble patriotism. Politicians are not our only patriots. There are many who love their country with a crowning devotion who do not manifest it in a political career, and it has come to pass in American politics that he who loves his country best, deals not in politics. In the highest, best sense of the word, let the physician be a patriot. He has as many causes as any other man to love his country, and many of them have more—those who live in a *sickly neighborhood.* We, of the Sunny South, notwithstanding the bad fortune of the late war, yet have the best country in the world. To her sons, her hills and dales, her flowers and fruits, her developments, and *her daughters*, should be more attractive than all else beside.

> " Lives there a man with soul so dead
> Who never to himself hath said,
> This is my own, my native land?"

> " Where'er I am, whatever realm I see,
> My heart untrammeled fondly turns to thee.
> I love thee next to Heaven above—
> Land of my fathers, thee I love!
> Then let them slander as they will,
> With all thy faults, I love thee still."

But, ladies and gentlemen, propriety suggests that I should not further occupy your time. And yet, I am loth to say farewell to these brave sons and fair daughters of Atlanta. Our stay in your midst has indeed been pleasant, and your kindness finds appreciation in our hearts. We will leave you your friends and admirers. We are proud of you as the people of our capital. We are not so narrow minded as to be envious of your prosperity, for it is ours; the glory of the head is the glory of the members. We shall watch your development and progress with delight, and expect you to become the mistress of the South. In taking leave of you, we have but one request to proffer. The interests of our Alma Mater are largely in your hands. Our farewell word is, let her share in your blessings.

To you, honored professors, it is sad to say farewell. True, it is not such a painful reflection that the headaches of the lecture room, the slight sensation of first experience in the dissecting room, and the terrors of the "green room," are all over; but we cannot leave your faithful tutorage and resign your personal advice and counsel without deepest regret. We would have you know that whatever measure of success we meet in life, we shall owe much of it to you. We fain would feel that we go hence retaining that interest you have so kindly taken in us.

Our wish is that your health may be good, your lives spared long, and your labors crowned with that success they so richly deserve.

For you, my comrades, I have reserved the last sad farewell. Our association has been most delightful. When your names are seen in the future, memory will gather around them all the halcyon recollections of these happy days. May the future hold in store for you the full fruition of your fondest hopes. In your unremitting toils, may you be regaled by the opening flowers of success and the full fruit of your best efforts. In the day of adversity put on indomitable perseverence; in times of success work as if adversity drove you to the task. Be never afraid to do right; fear nothing but your God and to do wrong. And may you, one and all, be soon cured of the multiplied ills of single-blessedness, in this life, and in the world to come, may you find a home where there are no *doctors*, is the farewell wish of your unworthy Valedictorian.

